# Association of a common TLR-6 polymorphism with coronary artery disease – implications for healthy ageing?

**DOI:** 10.1186/1742-4933-10-43

**Published:** 2013-10-30

**Authors:** Lutz Hamann, Alexander Koch, Saubashya Sur, Nadja Hoefer, Christiane Glaeser, Susanne Schulz, Michael Gross, Andre Franke, Ute Nöthlings, Kai Zacharowski, Ralf R Schumann

**Affiliations:** 1 Institute for Microbiology and Hygiene, Charité University Medical Center, Hindenburgdamm 27, 12003 Berlin, Germany; 2 Department of Anaesthesiology, Intensive Care Medicine and Pain Therapy, University Hospital, Theodor-Stern-Kai 7, 60590 Frankfurt, Germany; 3 Institute of Human Genetics and Medical Biology, University of Halle, Magdeburger Str. 2, 06112 Halle, Germany; 4 University School of Dental Medicine, Department of Operative Dentistry and Periodontology, Martin-Luther University Halle-Wittenberg, Germany Harz 42a, 06108 Halle, Germany; 5 Department of Cardiology, Charité University Medical Center, Hindenburgdamm 30, 12003 Berlin, Germany; 6 Institute for Clinical Molecular Biology, Christian-Albrechts-University of Kiel, Schittenhelmstrasse 12, Kiel, Germany; 7 PopGen Biobank, Institute for Experimental Medicine, Christian-Albrechts-University Kiel, Niemansweg 11, 24105 Kiel, Germany; 8 Current address: Department of Nutrition and Food Sciences, University of Bonn, Endenicher Allee 11-13, 53115 Bonn, Germany

**Keywords:** Coronary artery disease (CAD), Restenosis, Toll-like receptor (TLR) 6, Gene polymorphism, Innate immunity

## Abstract

**Background:**

The pro-inflammatory status of the elderly triggers most of the age-related diseases such as cancer and atherosclerosis. Atherosclerosis, the leading cause world wide of morbidity and death, is an inflammatory disease influenced by life-style and genetic host factors. Stimuli such as oxLDL or microbial ligands have been proposed to trigger inflammation leading to atherosclerosis. It has recently been shown that oxLDL activates immune cells via the Toll-like receptor (TLR) 4/6 complex. Several common single nucleotide polymorphisms (SNPs) of the TLR system have been associated with atherosclerosis. To investigate the role of TLR-6 we analyzed the association of the TLR-6 SNP Pro249Ser with atherogenesis.

**Results:**

Genotyping of two independent groups with CAD, as well as of healthy controls revealed a significant association of the homozygous genotype with a reduced risk for atherosclerosis (odds ratio: 0.69, 95% CI 0.51-0.95, *P* = 0.02). In addition, we found a trend towards an association with the risk of restenosis after transluminal coronary angioplasty (odds ratio: 0.53, 95% CI 0.24-1.16, *P* = 0.12). In addition, first evidence is presented that the frequency of this protective genotype increases in a healthy population with age. Taken together, our results define a role for TLR-6 and its genetic variations in modulating the inflammatory response leading to atherosclerosis.

**Conclusions:**

These results may lead to a better risk stratification, and potentially to an improved prophylactic treatment of high-risk populations. Furthermore, the protective effect of this polymorphism may lead to an increase of this genotype in the healthy elderly and may therefore be a novel genetic marker for the well-being during aging.

## Background

The immune status of the elderly is characterized by 2-4-fold increased baseline levels of inflammatory mediators such as cytokines and acute phase proteins [1]. This chronic low-level inflammation (recently also termed inflamm-aging) has been suspected to drive age related diseases such as atherosclerosis, cancer, diabetes and Alzheimer’s disease, all of them characterized by chronic inflammation [2-4].

Coronary artery disease (CAD) is the leading cause of morbidity and mortality worldwide [5]. It is an inflammatory disease with inflammatory markers being elevated, and causally involved in the pathogenesis of CAD [6]. CAD results in dangerous plaques leading to stenosis of coronary arteries, which are removed by a medical procedure termed percutaneous transluminal coronary angioplasty (PTCA). Restenosis, which is the main drawback of this treatment, has also been associated with increased local inflammation. The inflammatory response induced by PTCA correlates with the risk of restenosis, and anti-inflammatory treatments decrease the risk of restenosis [7-10].

In addition to life-style and nutrition, genetic factors have recently been shown to play an important role in the development of CAD. Several genetic risk loci have been identified by genome-wide association studies [11]. In line with these results, the release of inflammatory markers such as IL-6 during CAD, or following PTCA differs largely between individual patients and this most likely due to a different genetic background. Single nucleotide polymorphisms (SNPs) have been shown to influence serum levels of IL-6 and other inflammatory markers as reviewed by Dahmer et al. [12]. Although several genome-wide association studies have been performed, the loci identified could only explain some of the heritability of CAD and other common complex diseases. Therefore, further analysis of SNPs in candidate genes may improve our understanding of complex diseases such as atherosclerosis [13].

Postulated mediators of atherogenesis, e.g. bacteria such as *Chlamydia pneumoniae* or oxLDL, activate the innate immune system via the TLR-pathway [14]. TLR-associated SNPs have been found to be potentially involved in altered susceptibility and course of several diseases [15]. I.e.TLR-4 has been shown to be involved in early atherosclerosis by comparing knock out mice to control animals: Mice lacking TLR-4 were protected in an atherosclerosis model, which seemed to be independent from cholesterol levels [16]. In line with these findings, genetic variations within the TLR-4 gene in patients have been associated with risk for atherosclerosis, although conflicting results have been published (reviewed by den Dekker et al. [17]). Also deficiency of TLR-2 has been associated with lowered diet- and pathogen induced atherosclerosis in mouse models [18,19]. Involvement of the activation of the innate immune system via the TLR-pathway in pathogenesis of atherosclerosis as well as TLRs as possible therapeutic targets for atherosclerosis has been recently reviewed [20-22].

TLR-6 has been shown to mediate inflammatory responses upon stimulation with oxidized low-density lipoprotein (oxLDL) in cooperation with TLR-4 [23]. In line, TLR-4- and TLR-6-knock out mice both have been shown to express decreased quantities of tissue factor induced by hypercholesterolemic diet as compared to control mice [24]. Furthermore, the TLR-6 SNP Pro249Ser has recently been associated with lower left ventricular wall thickness and inflammatory response in hypertensive women [25]. In addition, the mutated TLR-6 Ser allele has been shown to lead to a diminished IL-6 release upon stimulation with a TLR-6 agonist [26]. Regarding restenosis, TLR-2 the heterodimerization partner of both, TLR-1 and TLR-6 in recognizing bacterial lipoproteins, has been shown to play an important role in restenosis after revascularization procedures: We could show that a TLR2 SNP is associated with the inflammatory response and with the risk of restenosis after PTCA [27].

Here we investigated the association of the TLR-6 SNP Pro249Ser (rs5743810) with susceptibility to atherogenesis in two independent patient groups with proven coronary artery disease by comparison with age-matched healthy control groups. One group that underwent PTCA and subsequent control angiography after 6 months was further analyzed for an association of the TLR-6 SNP with restenosis. We found the homozygous mutated TLR-6 genotype to be significantly associated with a reduced susceptibility to atherosclerosis (odds ratio of 0.69, 95% CI 0.51-0.95; *P* = 0.02). However, association with restenosis after successful PTCA was found to be only of borderline significance (odds ratio: 0.53, 95% CI 0.24-1.16, *P* = 0.12), which is most likely due to the lower sample number. Comparison of both aged healthy control groups with a young healthy control group showed a significant increase (odds ratio 1.53, 95% CI 1.16-2.02, *P* = 0.003) of the homozygous mutated genotype with age.

## Results

### TLR-6 SNP Pro249Ser is associated with the risk for atherosclerosis

207 Caucasian patients with symptomatic CAD as well as a confirmatory study group of 306 patients that underwent cardiac surgery were genotyped for the presence of the TLR-6 SNP Pro249Ser. Two groups of 300, and 305, respectively healthy Caucasian volunteers served as controls. Compared to healthy controls, the Ser allele was found to be significantly less frequent in patients (*P* = 0.008 and *P* = 0.23, respectively, Table 1). Combination of both cohorts resulted in a *P*-value of 0.007. Genotype distribution was also significantly different among patients and controls: Employing a recessive model we compared homozygous mutant genotypes with wild-type plus heterozygous genotypes: In both groups a trend for a decreased frequency of the homozygous genotype could be observed in patients indicating a protective function of this genotype (Table 1). Combination of both groups revealed a decreased risk for the homozygous Ser/Ser genotype with an odds ratio of 0.69, 95% CI 0.513-0.953 with a *P*-value of 0.02 (Table 1).

**Table 1:** TLR-6 genotype distribution in CAD patients and controls

	**N**	**Variant allele (%)**	***P*-value**	**Genotype (%)**			**Odds ratio (95% CI)**
				**Pro/pro**	**Pro/ser**	**Ser/ser**	
							***P*-value**
		**ser**		**C/C**	**C/T**	**T/T**	
**Study group**							
Controls 1	300	278 (46.3)	**0.008**	87 (29.0)	148 (49.3)	65 (21.7)	0.64
Patients	207	157 (37.9)		81 (39.1)	95 (45.9)	31 (15.0)	0.38-1.02
							0.06
**Confirmatory group**							
Controls 2	305	268 (43.9)	0.23	97 (31.8)	148 (48.5)	60 (19.7)	0.76
Patients	306	248 (40.5)		106 (34.6)	152 (49.7)	48 (15.7)	0.50-1.15
							0.20
**Combined**							
Controls	605	546 (45.1)	**0.007**	184 (30.4)	296 (48.9)	125 (20.7)	**0.69**
Patients	513	405 (39.5)		187 (36.5)	247 (48.1)	79 (15.4)	**0.513-0.953**
							**0.02**

*P*-values were determined by chi^2^ test. The odds ratios were determined by comparing the homozygous genotype with the wild type and the heterozygous genotype.

### Presence of the Pro249Ser TLR-6 SNP is associated with a trend towards the risk for restenosis

The risk of restenosis after successful PTCA has been associated with an inflammatory response induced by PTCA. Therefore, we stratified our study group for the occurrence of restenosis as determined by a control angiography 6 month after successful PTCA. In line with the previous finding, the TLR-6 Ser allele was also underrepresented in the restenosis group. However, these differences were of borderline significance only (*P* = 0.05), most likely due to the low sample number. A trend for a protective role of the homozygous Ser/Ser genotype regarding restenosis, Odds ratio 0.53, 95% CI 0.24-1.16, *P*-value = 0.12 was observed (Table 2).

**Table 2:** TLR-6 genotype distribution among CAD patients with or without restenosis

	**N**	**Variant allele (%)**	***P*-value**	**Genotype (%)**			**Odds ratio (95% CI)**
				**Pro/pro**	**Pro/ser**	**Ser/ser**	***P*-value**
		**Ser**		**C/C**	**C/T**	**T/T**	
**Study group**							
No restenosis	106	90 (42.5)	0.05	36 (34.0)	50 (47.2)	20 (18.9)	0.53
Restenosis	101	67 (33.2)		45 (44.6)	45 (44.6)	11 (10.9)	0.24-1.16
							0.12

*P*-values were determined by chi^2^ test. The odds ratios were determined by comparing the homozygous genotype with the wild type and the heterozygous genotype.

### Frequency of the Pro249Ser TLR-6 SNP increases with age in healthy controls

Given that coronary artery disease is the major cause of death in the Western world, we speculated that this protective Ser249Ser TLR-6 genotype would be overrepresented in an elderly cohort of healthy controls in comparison to a young healthy control group, where due to age CAD doesn’t play a role. To test this hypothesis, we compared 805 young healthy controls (age <50) with both combined aged healthy control groups of higher age described above. Indeed we observed a significant increase of the “protective Ser allele” in the high age control group (P = 0.00006). In line, the homozygous mutated Ser/Ser genotype also was found more frequently in higher age (Odds ratio 1.53, 95% CI 1.16-2.02, P = 0.003, Table 3) indicating this genotype to be a marker for healthy ageing. Comparing genotype and gender by chi^2^ test no significant association could be observed for patients, old controls and young controls, respectively (*P* = 0.33, *P* = 0.94, *P* = 0.72).

**Table 3:** TLR-6 genotype distribution among young and old healthy controls

	**N**	**Variant allele (%)**	***P*-value**	**Genotype (%)**			**Odds ratio (95% CI)**
				**Pro/pro**	**Pro/ser**	**Ser/ser**	
							***P*-value**
		***P***	**value**	***P***	**value**	***P***	
**Study group**							
Young contr.	805	606 (37.6)	**0.00006**	316 (39.3)	372 (46.2)	117 (14.5)	**1.53**
							**1.16-2.02**
							**0.003**
Old contr.	605	546 (45.1)		184 (30.4)	296 (48.9)	125 (20.7)	

*P*-values were determined by chi^2^ test. The odds ratios were determined by comparing the homozygous genotype with the wild type and the heterozygous genotype.

### Possible influence of Pro249Ser on TLR-6 protein structure

**Figure 1:**
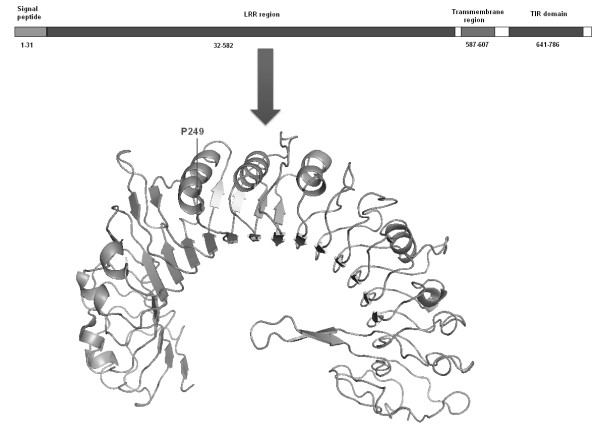
**3D structure of Leucine rich region (LRR) of TLR-6 housing the SNP P249S.** Horizontal bar shows distribution of different regions in TLR6 protein (796aa). Numbers indicate amino acid residues for a particular region. Blocks in white are amino acid residues not associated with any region.

To investigate the consequence of the Pro249Ser SNP on TLR-6 protein structure we performed some “*in silico* analysis”. Figure 1 shows three-dimensional structure of Leucine rich region (LRR) for TLR-6 housing the SNP. Comparison of CASTp results for wild type and mutant of TLR-6 LRR region divulged presence of 102 and 93 functional pockets, respectively (Additional file 1: Figure S1). The mutation resulted in a decrease number of pockets. The wild type and mutant showed variation in surface topography of major clefts and cavities (Additional file 2: Figure S2). As seen from Additional file 3: Table S1 and Additional file 2: Figure S2, significant decrease in volume of two major clefts in the mutant was observed. Flexibility analysis revealed that the wild type was 36.6% rigid while mutant was 70.7% rigid. I-Mutant results predicted DDG value (difference in free energy of the mutation) of −1.50 Kcal/mol owing to change of P249 to S249.

## Discussion

Inflammation plays a pivotal role in atherosclerosis and mediates the effects of many known risk factors of the disease by altering the behaviour of endothelial as well as smooth muscle cells [28]. In addition, the risk for restenosis after successful angioplasty is strongly influenced by the inflammatory response to the PTCA procedure, mediated at least partially by the TLR system triggering the inflammatory response [7,9].

Here, we add another line of evidence for the involvement of the TLR system in this inflammatory process leading to atherogenesis. TLR-6 has been shown to mediate inflammatory response by oxLDL, a known risk factor for atherosclerosis [23]. Bacterial lipopeptides, also known to induce atherogenesis, are also recognized by TLR-6 in combination with TLR-2 and TLR-1 [14]. Our results shown here indicate an association of the TLR-6 Pro249Ser SNP with the risk for atherosclerosis, and potentially with the risk for restenosis after successful PTCA. The reason for the association with restenosis being only of borderline significance most likely lies in the limited sample number since restenosis data were not available for the confirmatory study group. However, the differences in percentage were similar or even more pronounced as found by the comparing patients and controls regarding atherosclerosis susceptibility. Limitations of our study are on one hand the low sample number, and on the other the fact, that little information about other relevant risk factors potentially influencing our results was available. Therefore, larger and well planned prospective studies are needed to confirm our results.

In line with our results several mouse models have currently shown an involvement of TLR-2, which acts as heterodimer with TLR-1 or −6, in atherogenic processes [18,19]. It has been suggested that endogenous TLR-2 agonists may have an impact on atherosclerotic disease progression by the fact that TLR-2 knock out mice exhibit a decreased severity of experimentally induced atherosclerosis [19]. Two studies have shown pro-atherogenic effects of apoCIII being mediated by TLR-2: In contrast to wild type mice apoCIII failed to activate monocytes originating from TLR-2 deficient mice regarding adhesiveness to HUVECs [29]. Furthermore, apoCIII was not able to induce MCP-1 and IL-6 in adipose tissue from TLR-2 knock out mice [30].

Elevated IL-6 levels play an important role in atherogenesis due to detrimental effects on the vascular wall and endothelial function [31,32]. Supporting our hypothesis of TLR-6 Pro249Ser being athero-protective functional analysis of this SNP has revealed that the Ser/Ser genotype was associated with a decreased IL-6 release by stimulated monocytes as well as a lessened NF-κB activation in transfected HEK 293 cells [26]. In contrast to IL-6, the release of the athero-protective cytokine IL-10 seems not to be influenced by TLR-6 Pro249Ser [33,34]. It has furthermore been shown that murine aortae express TLR-6 and that it is crucial for the induction of vascular dysfunction by Gram-positive bacteria [35]. The role of TLR-6 deficiency in atherogenesis has recently investigated employing knock out mice. Endogenous atherosclerotic stimuli brought about by high fat diet, for example oxLDL, do not depend on TLR-6 expression, whereas exogenous stimuli like MALP2, known to be a TLR-6 ligand, depend on TLR-6 expression [36]. However, whether the TLR-6 Pro249Ser SNP alters the inflammatory response in human vascular cells and which stimuli are involved currently still remains to be investigated. The therapeutic potential of TLR modification by TLR agonists as well as TLR antagonists in cardiovascular dysfunction has been reviewed recently. Especially compounds modifying TLR-2 and TLR-4 function are considered to be beneficial. Antagonists are thought to lower the inflammatory burden whereas low concentrations of agonist are thought to be protective in terms of preconditioning [37]. Our data indicate that also TLR-6 could be a target of therapeutical intervention.

The consequence of the mutation on the protein structure is evident from our findings. In wild type, the pocket housing SNP P249S showed differences having residues interrupted by cavities in proximity of proline while mutant had pocket residues close to each other indicating potential conformational changes. This conformational change is likely to affect binding of ligands and receptors. Again, substantial decrease in volume of the clefts is bound to impact capability of mutant protein to enter into meaningful interactions since majority of binding regions and active sites are known to be located in largest cleft [38]. These aforementioned facts are further supported by the outcome of flexibility studies. The wild type protein being more flexible had more room for ligand induced movements while in mutant ligand induced movement will be restricted to side-chain rearrangements only [39]. So, the mutation makes the structure difficult to undergo functionally relevant conformations making it harder for inducing possible drug molecules. The significantly low DDG value pointed out considerable decrease in stability in the mutant. All these confirmed that the TLR-6 protein structure and functionality are influenced by mutation.

Healthy aging depends most likely on the delay of onset of age related diseases such as coronary artery disease, Alzheimer’s disease, type 2 diabetes and cancer [40]. Genetic variations associated with age-related diseases therefore have been suspected to be involved in successful aging [41]. A large number of genes, and thereby a large number of SNPs, are involved in pathogenesis of these common diseases. Therefore, a genetic signature for longevity has been suggested by Sebastiani et al. [42]. They established networks for coronary artery disease and Alzheimer’s disease including 130 disease-associated genes building a genetic risk model [42]. However, since TLR-6 SNPs have not been associated with either of these diseases so far, TLR-6 was not included into this model. Of note, since chronic inflammation is central for age-related diseases these longevity networks are both centred on NFκB activation [42], which is the main signalling pathway following TLR stimulation. Having shown that the TLR-6 ser/ser genotype protects from atherosclerosis we could show here that this genotype is increased in the healthy elderly. One could speculate that this less functional TLR-6 variant may be beneficial by lowering the effects of inflamm-ageing during older age.

## Conclusion

Our results suggest that the less functional TLR-6 Ser/Ser genotype protects from atherosclerosis as well as from restenosis after successful PTCA. The underlying mechanism remains to be investigated but it is tempting to speculate that a lessened inflammatory response brought about by a dysfunctional TLR-6 is responsible for this protective effect. Genotyping of patients at risk for CAD may improve the risk stratification and lead to a better prevention regime for both, atherosclerosis and restenosis. Furthermore, this SNP may be a novel genetic marker for healthy aging.

## Methods

### Patients

#### Study group

The first study group has been described in detail previously as study group “A” [27]. In brief, the group consisted of 207 Caucasian patients with symptomatic coronary artery disease who underwent PTCA. Significant stenosis was confirmed by quantitative coronary angiography. All patients were hospitalized, clinically stable, and were routinely checked after 6 month for follow-up angiography.

#### Confirmatory group

The confirmatory group has been described previously [43]. 306 patients undergoing elective cardiac surgery (coronary artery bypass graft with/or without valve surgery) have been enrolled by a single-center study.

#### Controls

DNA from 300 healthy controls obtained from the *PopGen* biobank (University of Kiel, Germany) was included as control for the study group (Control group 1). DNA from 305 healthy controls from the *PopGen* biobank was included as control for the confirmatory group (Control group 2). A third control group of 805 young healthy volunteers was analyzed to compare genotype distribution dependent on age (Control group 3). Participants reported to not have had a chronic disease or regularly use medication at recruitment.

Characteristics for all patients and controls are summarized in Table 4. All participants were of Caucasian origin. All studies were approved by the local ethics committees, and all investigations were carried out in accordance with the ethical guidelines of the 1975 Declaration of Helsinki.

**Table 4:** Patient characteristics

	**Study group**	**Confirmatory group**	**Controls 1**	**Controls 2**	**Controls 3**
Mean age, SD	59.8, 9.94	68.2, 9.0	55.5, 8.41	68.0, 9.08	30.2, 7.39
Male/female (%)	77/23	75/25	80/20	74/26	47/53
Diabetes (%)	9.8	45.0	0	0	0
Smoker (%)	25.0	nd	nd	nd	nd
Hypertension (%)	41.4	nd	0	0	0
BMI	27.3	27.8	nd	nd	nd
Procedure	nd	ACVB 82%			
		ACVB + VS 18%			

ACVB: aorto coronary vein bypass, VS: valve replacement, nd: not determined.

### Genotyping

Genomic DNA was prepared by standard procedures from whole blood. Genotyping for TLR-6 SNP Pro249Ser (rs5743810) was performed by melting curve analysis. Melting curve analysis was carried out employing primers gaaagactctgaccaggcat (forward), ctagtttattcgct atccaagtg (reverse), and FRET-hybridisation probes: accagaggtccaaccttactgaa-FL and LC-red 640-ttaccctcaaccacatagaaacgacttgga resulting in melting points of 61°C and 52°C for the wild-type,, and the mutated allele, respectively. Genotyping was executed using standard PCR conditions applying the LightCycler LC 480 platform (Roche Diagnostics, Mannheim, Germany). Primers and probes were designed by O. Landt (TIB-MOLBIOL, Berlin, Germany). Genotyping by melting curve analysis was validated by reanalyzing 10% of the samples using a conventional restriction fragment polymorphism analysis. Digestion of the 194 bp PCR product with AvaII results in two fragments of 67 bp and 127 bp in wild type individuals, whereas the TLR-6 SNP Pro249Ser destroys this restriction site. Restriction digests were analyzed on a 3% agarose gel. No divergent results were observed.

### Hardy weinberg equilibrium

All control groups were analyzed for Hardy Weinberg equilibrium (HDW). Deviations from HDW could not be detected (control group 1: *P* = 0.89, control group 2: *P* = 0.79, control group 3: P = 0.44).

### Protein structure analysis

The structure of Leucine rich region (LRR) of TLR-6 was determined by homology modelling technique using MODELLER 9.11 program [44]. Owing to lack of homology and availability of suitable templates, structure of other regions could not be determined. Swiss-Pdb Viewer [45] was used to perform energy minimizations. Structures were visualised with PyMol (http://www.pymol.org). Algorithms ProSA [46], PROCHECK [47], PROVE and ERRAT (http://nihserver.mbi.ucla.edu/SAVS/) were used for structure evaluations. Surface topography and pockets were determined with CASTp [48]. These pockets contributed towards functioning and understanding properties [48]. Clefts and cavities were predicted using ProFunc [49]. These regions house possible functional biologically important residues. The area and volume of clefts are significant for interacting residues, ligand binding, receptor binding etc. [38]. Flexibility studies were based on the software StoneHinge [50]. The implication of mutation on structure was studied using I-Mutant 3.0 [51]

### Statistics

*P*-values for genotype associations were determined by chi^2^ test. Risk factors were estimated by logistic regression analysis adjusting for age and sex using SPSS Statistics 19 software.

## Competing interests

The authors declare they have no competing interests.

## Authors’ contributions

LH: Study design, analysis of patients DNA, writing the manuscript; AK and KZ: Sampling and characterisation of CAD patients (confirmatory group); SS: Protein modelling; NH: Analysis of young healthy controls; CG, SS and MG: Sampling and characterisation of CAD patients (study group); AF and UN: Providing PopGen control DNAs; RS: study design, critical reading, writing and discussion of the manuscript. All authors read and approved the final manuscript.

## Supplementary Material

Additional file 1: Figure S1 Pockets for A) wild type and B) mutant of human TLR6 LRR region. Pocket housing the SNP is marked red. Click here for file

Additional file 2: Figure S2 Major clefts and cavities for wild type and mutant of human TLR6 LRR region. Click here for file

Additional file 3: Table S1 Comparison of volume of 4 major clefts for wild type and mutant protein. Click here for file
