# Supplementary figures and images for: Association of a common TLR-6 polymorphism with coronary artery disease – implications for healthy ageing?
Source: Immun Ageing. 2013 Oct 30;10:43. doi: 10.1186/1742-4933-10-43 (PMC4028875; doi:10.1186/1742-4933-10-43)

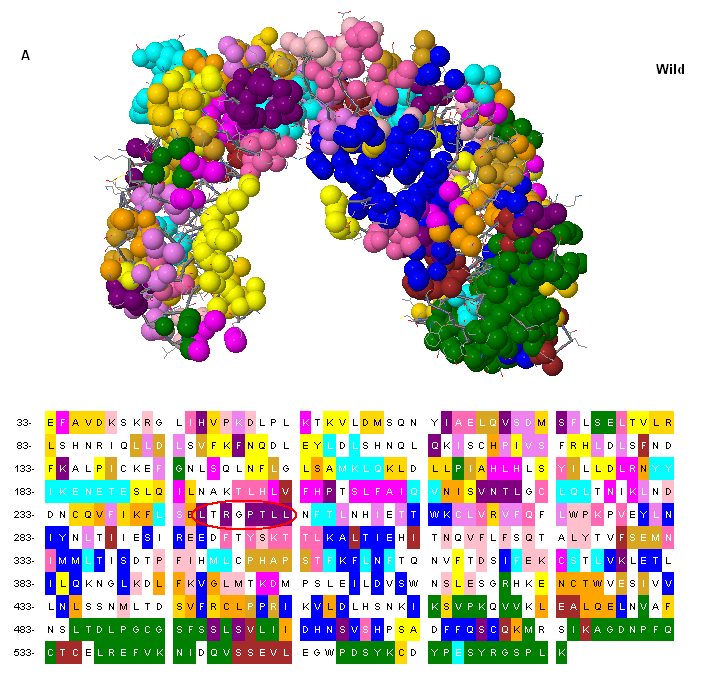

Supplement: Additional file 1: Figure S1 — Pockets for A) wild type and B) mutant of human TLR6 LRR region. Pocket housing the SNP is marked red. [file 1742-4933-10-43-S1.zip › 2324856701026615_add1a.tiff]

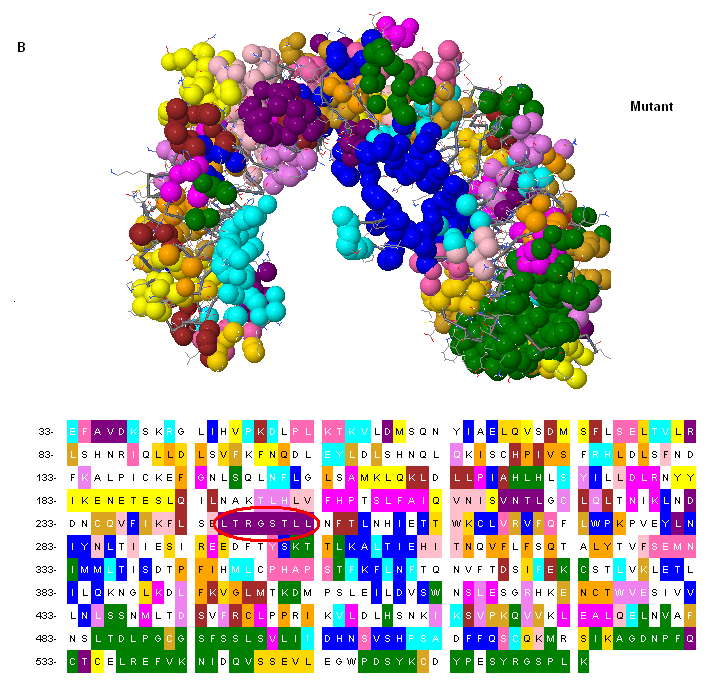

Supplement: Additional file 1: Figure S1 — Pockets for A) wild type and B) mutant of human TLR6 LRR region. Pocket housing the SNP is marked red. [file 1742-4933-10-43-S1.zip › 2324856701026615_add1b.tiff]

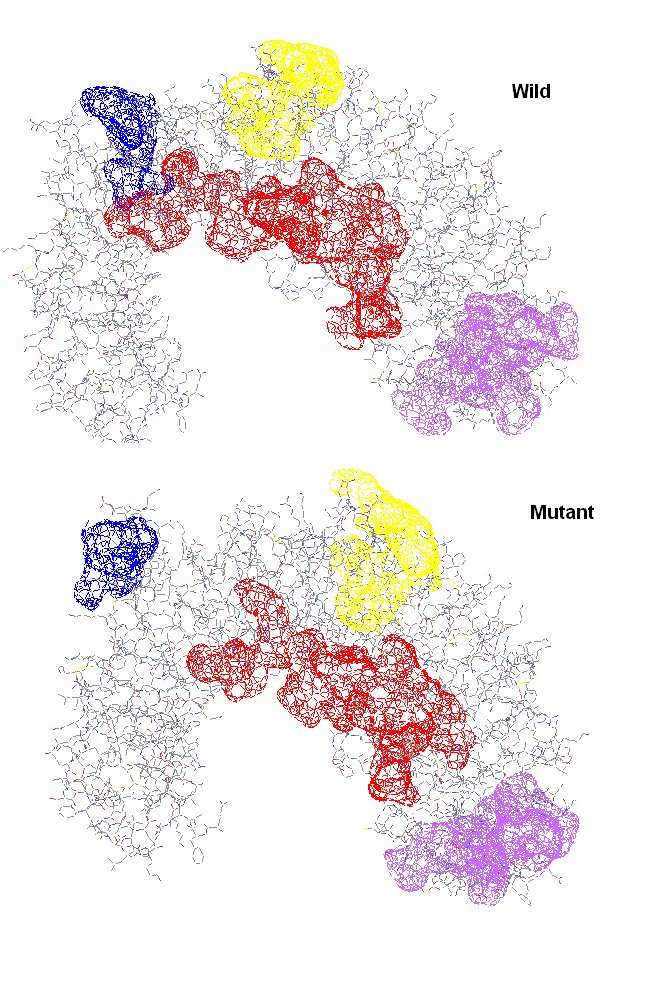

Supplement: Additional file 2: Figure S2 — Major clefts and cavities for wild type and mutant of human TLR6 LRR region. [file 1742-4933-10-43-S2.tiff]
