# Supplementary material for: Association of a common TLR-6 polymorphism with coronary artery disease – implications for healthy ageing?
Source: Immun Ageing. 2013 Oct 30;10:43. doi: 10.1186/1742-4933-10-43 (PMC4028875; doi:10.1186/1742-4933-10-43)
Supplement: Additional file 3: Table S1 — Comparison of volume of 4 major clefts for wild type and mutant protein. [file 1742-4933-10-43-S3.docx]

**Supplementary Table**

**Table S1**: Comparison of volume of 4 major clefts for wild type and mutant protein

| Clefts | Wild type  protein  [(Å³)](http://www.aqua-calc.com/what-is/volume/cubic-angstrom) | Mutant  protein  [(Å³)](http://www.aqua-calc.com/what-is/volume/cubic-angstrom) |
| --- | --- | --- |
| 1(in red) | 4580.30 | 4257.56 |
| 2(in pink) | 3238.75 | 3304.97 |
| 3(in yellow) | 1701.84 | 1716.61 |
| 4(in blue) | 1149.19 | 855.14 |
